# Influence of Long-Term Subcritical Annealing on the Unalloyed Steel Welded Joint Microstructure

**DOI:** 10.3390/ma16010304

**Published:** 2022-12-28

**Authors:** Dominika Fajt, Mariusz Maślak, Marek Stankiewicz, Paulina Zajdel, Krzysztof Pańcikiewicz

**Affiliations:** 1 Faculty of Metals Engineering and Industrial Computer Science, AGH University of Science and Technology, 30059 Cracow, Poland; 2 Faculty of Civil Engineering, Cracow University of Technology, 31155 Cracow, Poland

**Keywords:** spheroidizing, welded joint, structural steel

## Abstract

The article presents changes in the microstructure of hot-rolled unalloyed structural steel after the arc welding process and in the state after long-term exposure to 600 °C during operation. These studies enable a clear assessment of the effects of long-term exposure to elevated temperature relative to the as-welded condition, which has not been reported. The microstructure examination was carried out on welded joints in eight different zones of the joint. Studies have shown that the welding thermal cycle causes significant changes in the microstructure in the area of the base material heated above the A_1_ temperature—the heat-affected zone (HAZ)—and in the weld area in the case of multi-pass welding. The long-term exposure of the subcritical temperature of 600 °C on the welded joint leads to the phenomenon of cementite spheroidization in the pearlite in all zones of the joint, while preserving the band structure of the steel after rolling and the structural structure. In the case of the weld, acicular and side-plate ferrite disappearance was observed.

## 1. Introduction

Structural steels are used in many industries, from everyday products to large building structures. Due to their uncomplicated chemical composition and the use of conventional manufacturing processes for their production, they are characterized by good processing properties—deformability and weldability [1,2,3,4,5].

The banded ferritic-pearlitic microstructure of the steel obtained in the hot-rolled condition undergoes some controlled and uncontrolled changes due to thermal and mechanical influence [6,7,8]. In the case of inseparable joining of elements in welding processes, a new component is created that connects two materials—a weld—with a dendritic structure, the characteristics of which are similar to those of the cast material. The material joined in the weld area is characterized by changes caused by rapid heating to a temperature lower than solidus and subsequent cooling, which leads to the formation of a heat-affected zone. As they move away from the fusion line, individual areas are heated to lower and lower temperatures. Despite the relatively short time of staying at a given maximum temperature and fairly quick cooling, areas identical to those after ordinary heat treatment—annealing with phase transformation—are observed in the HAZ (heat-affected zone) of structural steels. The highest heated area—CGHAZ (coarse grain HAZ)—is characterized by a coarse-grained structure of the former austenite grain, similarly to that after homogenizing annealing. The area heated slightly above the temperature of the end of the eutectoid transformation—FGHAZ (fine grain HAZ)—is characterized by a fine-grained structure, similar to normalized steel. In the area heated in the range of intercritical temperatures A_1_ (which defining the lower limit of existence of austenite) and A_3_ (which defining the upper limit of existence of ferrite)—ICHAZ (intercritical HAZ)—the material undergoes partial grain refinement, i.e., in the area that has undergone recrystallization, similar to during incomplete annealing. Areas of hot-rolled steel heated to a subcritical temperature do not undergo any visible transformation [9,10,11], unlike martensitic steels, where an area softened due to tempering (over-tempered region) is observed [12].

Welded joints, due to changes in the microstructure or state of stress, are subjected to heat treatment, including annealing processes with transformation (e.g., normalization) and without transformation (stress relief annealing) [13,14]. The impact of elevated temperature may also be related to the operating conditions of welded structures, which mainly depends on their application. There are needs, such as boilers or reactors, in which the increased process temperature, below the critical temperature, affects the structure in a way analogous to the heat treatment without transformation [15,16]. An example of such a process is lead refining, which, depending on the type of pollutant to be removed, is carried out in various temperature ranges, even reaching 600 °C [17]. It can be expected that long-term operation in this temperature range will give effects that are observed, for example, after spheroidizing annealing.

**Figure 1:**
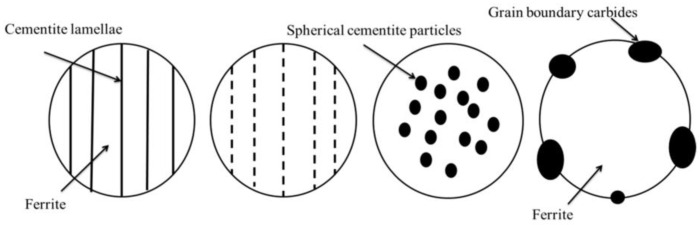
Schematic illustration of pearlite spheroidization. Reprinted from ref. [19].

ISO 4885 [18] defines spheroidizing as annealing just below the A_1_ temperature of steels with long soaking time to bring the carbides in the form of spheroids. The microstructure obtained in this process is spheroidite, defined as characteristic soft microstructure consisting of sphere-like globular cementite particles within a ferrite matrix. Wang et al. [19] presented a diagram of the progress of the spheroidization process of lamellar cementite in pearlite (Figure 1).

Although the ISO 4885 standard and other textbook heat treatment charts indicate a temperature range of spheroidizing annealing oscillating around the A1 temperature, there are a number of studies proving that this process also occurs at lower temperatures. Stodolny et al. [20] found a 100% content of spheroidite in C45 steel after annealing at 700 °C/1 h and 600 °C/23 h, and at 500 °C after 23 h an increase in the content of spheroidite of 11% was achieved. Wang et al. [19] conducted spheroidization in 14Cr1MoR steel at 680 °C for 22, 40, 70, and 100 h, observing the continuous progress of spheroidization, represented, among others, by a decrease in hardness from approximately 32.5 HRC to approximately 24 HRC after 100 h of annealing. Yang and Lu [21] observed a decrease in the mechanical properties of cold-rolled SCM435 steel after spheroidization at 700 °C/5 h and 680 °C/5 h, however, the observed effects are related to both carbide spheroidization and recrystallization of cold-deformed grains. Arruabarrena and Rodriguez-Ibabe [22] found a decrease in the hardness of fine pearlite with increasing annealing time at 720 °C, 660 °C, and 600 °C in AISI 5140 steel. On the basis of the studies mentioned above, it can be assumed that a very long exposure to temperatures below A_1_ may, in consequence, also lead to spheroidization of cementite, similar to during annealing around the A_1_ temperature.

As part of the research, the microstructure of the welded joint of unalloy steel in the as-welded condition and in the condition after long-term exposure at 600 °C was analysed. Long-term exposure (amounting to over 4000 h) is related to the operation of the product that contains the analysed welded joint at a temperature of 600 °C. The aim of the research is to assess the influence of the state of welded joints on their microstructures. These studies enable a clear assessment of the effects of long-term exposure to elevated temperature relative to the as-welded condition, which has not been reported.

## 2. Materials and Methods

**Figure 2:**
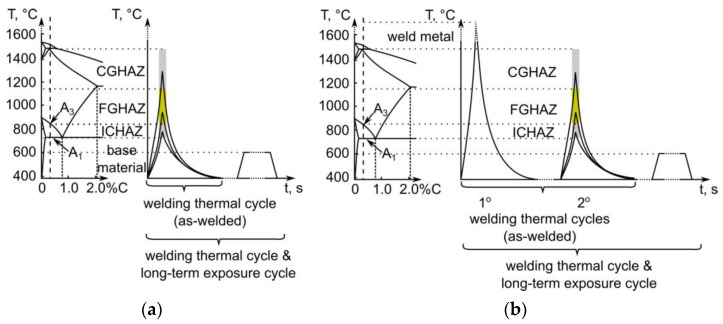
Characteristic zones analysed in the: (**a**) material; (**b**) weld metal.

The test materials are two gas metal arc-welded joints made of EN 10025-2: S235JR structural steel (Werkstoffnummer 1.0036; equivalent to A283 Grade C) with a thickness of 40 mm. The filler metal was EN ISO 14341-A: G 42 2 M G2Si. The first joint was tested in the as-welded condition, and the second was tested after long-term exposure (>4000 h) at 600 °C. Both joints were analyzed in all characteristic zones, which are schematically summarized in Figure 2. Observations were made in the base material and its HAZ (Figure 2a) and the weld metal and its HAZ, resulting from multi-pass welding (Figure 2b) in the as-welded condition and in the as-welded condition with subsequent long-term exposure.

The chemical compositions of the base material according to the EN 10025-2 standard, the filler metal according to the EN ISO 14341-A standard and Optical Emission Spectroscopy (OES) analysis are presented in Table 1.

The OES was performed with the Foundry Master-WAS Spectrometer (Hitachi, Tokyo, Japan). Observation of the microstructure was performed with a Leica DM/LM (Leica, Wetzlar, Germany) light microscope (LM) with a bright field (BF) and a Phenom XL (Thermo Fisher Scientific, Waltham, MA, USA) scanning electron microscope (SEM) with backscattered electrons (BSE). The SEM-BSE images were excited by an electron beam with an accelerating voltage of 20 kV and a current of 10 nA. For microscopic examination, the samples were etched in a 4% alcoholic nitric acid solution.

## 3. Results and Discussion

### 3.1. Microstructure of Base Materials

**Figure 3:**
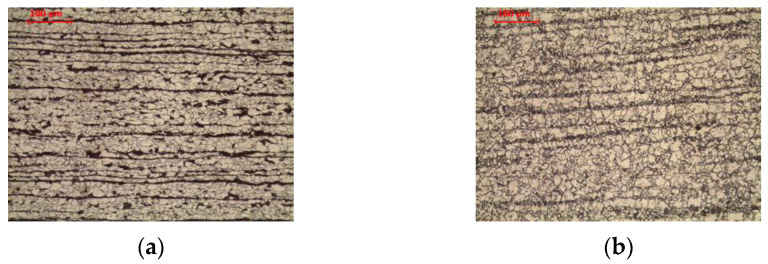
Microstructure of base material: (**a**) delivery state; (**b**) after long-term exposure at 600 °C, LM-BF.

**Figure 4:**
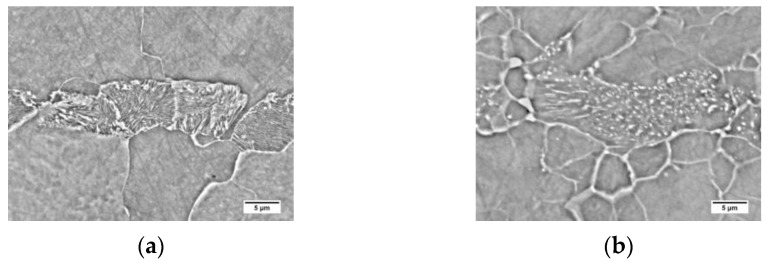
Microstructure of base material: (**a**) delivery state; (**b**) after long-term exposure at 600 °C, SEM-BSE.

The tested steel was characterized by a banded ferritic-pearlitic microstructure, characteristic of products shaped by hot rolling (Figure 3a). Allotriomorphic ferrite grains of various sizes were observed. Pearlite colonies, composed of alternating ferrite and cemenethite lamellae (Figure 4a), were arranged in bands in the direction of rolling. An analogous structure after hot rolling of non-alloy steel was observed, among others, by Chien-Cheng et al. [23].

Long-term exposure at 600 °C led to spheroidization of the cementite in the pearlite (Figure 3b). Globular precipitates of iron carbide in the ferritic matrix are observed (Figure 4b). Despite the change in the form of cementite, the band structure of steel was preserved. Matusiewicz et al. [24,25] observed an analogous perlite structure after annealing in a wide temperature range (620–700 °C).

### 3.2. Microstructure of Heat-Affected Zones

**Figure 5:**
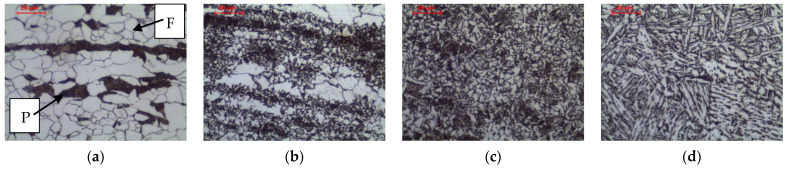
Microstructure of welded joint (as-welded): (**a**) base material; (**b**) intercritical HAZ; (**c**) fine grain HAZ; (**d**) coarse grain HAZ, LM-BF. Legend: F—ferrite, P—pearlite.

**Figure 6:**
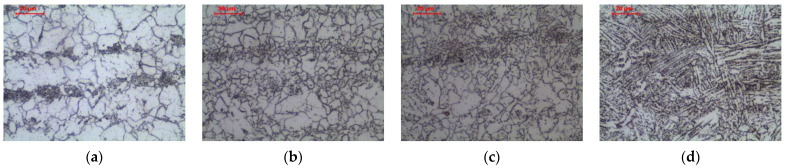
Microstructure of welded joint (as-welded and long-term exposure at 600 °C): (**a**) base material; (**b**) intercritical HAZ; (**c**) fine grain HAZ; (**d**) coarse grain HAZ, LM-BF.

The welding thermal cycle of welding significantly affects the microstructure of the base material, which is heated to different maximum temperatures depending on the distance from the fusion line. Figure 5 and Figure 6 show the microstructure of the base material and three basic zones in HAZ in as-welded state and after long-term exposure at 600 °C, which are described in detail below.

**Figure 7:**
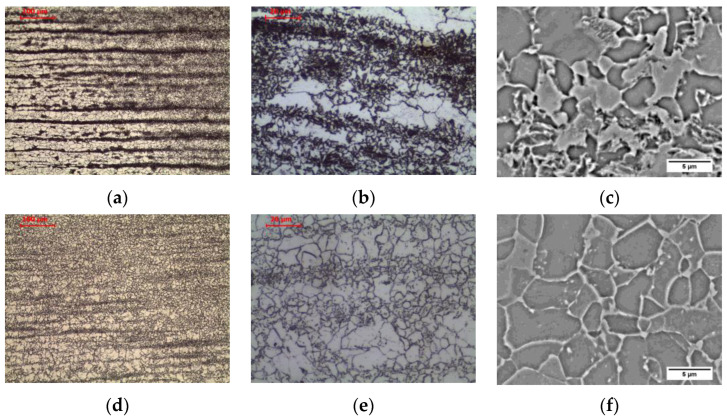
Microstructure of intercritical heat-affected zone: (**a**–**c**) as-welded state; (**d**–**f**) after long-term exposure at 600 °C, LM-BF, and SEM-BSE.

The material in intercritical area (ICHAZ), heated between temperatures A_1_ and A_3_ (Figure 5b and Figure 7a–c), retained its banded ferritic-pearlitic microstructure, but was partially recrystallized (Figure 7a–c). In the intercritical region ferrite and austenite coexist in equilibrium. Therefore, parts of the pearlite and ferrite undergo eutectoid transformation into austenite. Due to the short residence time in this temperature range, the nucleus of new grains does not experience a large growth. After cooling, a microstructure consisting of allotriomorphic ferrite with different grain sizes and pearlite with preserved structure banding was observed. Analogous effects were observed, e.g., Li et al. [26] in low carbon steel submerged arc welded and Yamamoto and Ito [27] in HT490 steel welded by gas metal arc welding and tungsten metal arc welding.

Long-term exposure of ICHAZ at 600 °C led to a change in the form of cementite in the pearlite (Figure 6b and Figure 7d–f). Globular precipitates of iron carbide in the ferritic matrix in the band of the former pearlite were observed. Despite the change in the form of cementite, the band structure of steel was preserved. The spheroidization of perlite in ICHAZ was observed, among others, by Khan and Sengfu [28] in the electroslag welded joint of the U71Mn pearlitic rail steel and Porcaro et al. [29] in the flash welded joint of the TR57 pearlitic rail steel. This phenomenon was caused by the occurrence of spheroidization due to slow cooling after welding from the area heated to a temperature between A_1_ and A_3_. Porcaro et al. [29] confirmed the spheroidization of cementite in dilatometric tests after heating to an intercritical temperature of 740 °C. 

**Figure 8:**
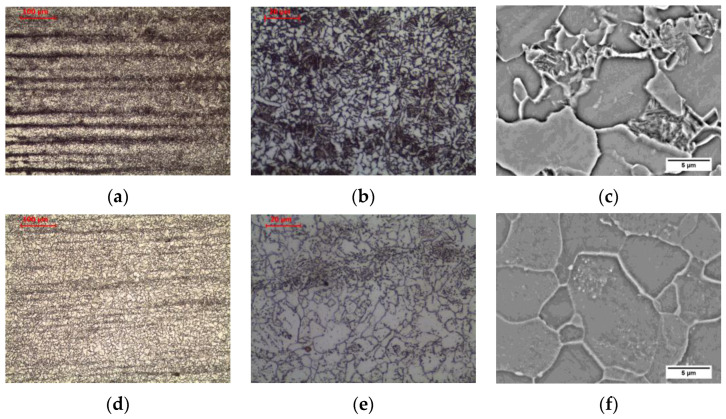
Microstructure of fine-grain heat-affected zone: (**a**–**c**) as-welded state; (**d**–**f**) after long-term exposure at 600 °C, LM-BF, and SEM-BSE.

The material in the fine-grained area (FGHAZ), heated above the temperature A_3_ but not exceeding 1150 °C [30], is characterized by a typical steel structure after normalization. The material heated above the A_3_ temperature undergoes complete recrystallization of ferrite and pearlite by means of eutectoid transformation into austenite. Due to the relatively low temperature, many nuclei of new austenite grains are formed, which, while growing, begin to block each other without excessively increasing the grain size. After being cooling to ambient temperature, the ferritic-pearlitic microstructure of fine-grained austenite formed as a result of the eutectoid transformation is characterized by fine grain (Figure 5c and Figure 8a–c). Relatively low temperature does not allow for redistribution of carbon due to diffusion, and hence the privileged places for the transformation of austenite into pearlite are areas enriched in carbon. This still preserves the band structure in this zone. This is confirmed by the observations of Yamamoto and Ito [26] in FGHAZ in HT490 steel and Jang et al. [31] in FGHAZ in AH36 steel. After cooling, a microstructure consisting of allotriomorphic ferrite with a relatively small grain size and pearlite with preserved structure banding was observed.

Long-term exposure of FGHAZ at 600 °C, as in the previous zone, led to a change in the form of cementite in pearlite (Figure 6c and Figure 8d–f). Globular precipitates of iron carbide in the ferritic matrix in the band of the former pearlite were observed. Long-term annealing did not affect the loss of the band arrangement of the structure components. This was confirmed by the observations of Guk et al. [32] on 16MnCrS steel, which was normalized before spheroidization. None of the processes deprived the steel of the band structure.

**Figure 9:**
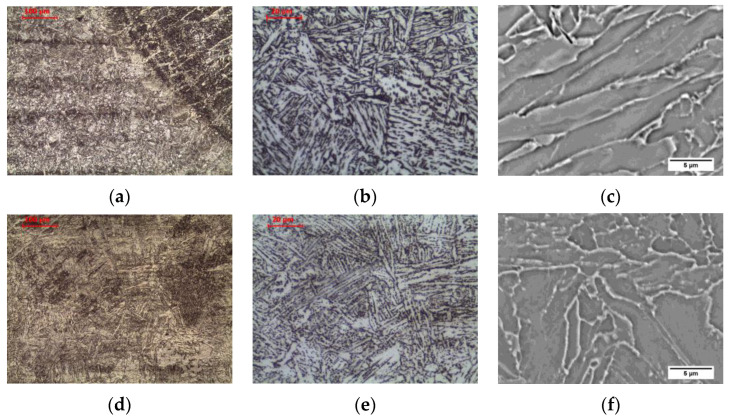
Microstructure of coarse-grain heat-affected zone: (**a**–**c**) as-welded state; (**d**–**f**) after long-term exposure at 600 °C, LM-BF, and SEM-BSE.

The material in coarse-grained area (CGHAZ), heated well above the temperature A_3_, exceeding 1150 °C [30], up to the solidus temperature, was characterized by a typical steel structure after homogenization or coarse grain annealing (Figure 5d and Figure 9a–c). As in the fine-grained zone, the material heated above the A_3_ temperature undergoes complete recrystallization of ferrite and pearlite by eutectoid transformation into austenite. However, due to the relatively high temperature, relatively fewer nuclei of new austenite grains are formed, which grow to large sizes. After being cooling to ambient temperature, the ferritic-pearlitic microstructure formed as a result of the eutectoid transformation is characterized by a large size of the former austenite grain (Figure 9a–c). The large grain of former austenite, by reducing the surface area of grain boundaries for ferrite nucleation, favours a small amount of allotriomorphic ferrite grains in favour of side-plate ferrite in the state after cooling to ambient temperature. At lower magnification, the band structure is still visible, which is the result of a too-short residence time in the maximum temperature range, making it impossible to homogenize the chemical composition. After cooling, a microstructure consisting of side-plate ferrite with a relatively large grain size of the former austenite and pearlite with preserved structure banding was observed. Zhang et al. [33] showed the appearance of a similar structure in simulated CGHAZ in HSLA steel over a wide range of cooling times. Similarly, Prado et al. [34] found the effects described above in CGHAZ micro-alloyed steel with addition of vanadium and niobium, and Jeong and Han [35] observed these effects in CGHAZ of C-Mn steels with varying aluminium content over a wide range of cooling rates.

Long-term exposure of CGHAZ at 600 °C, similar to the previous zones, led to a change in the form of cementite (Figure 6d and Figure 9d–f). Globular precipitates of iron carbide in the ferritic matrix were observed. The side-plate ferrite was preserved.

### 3.3. Microstructure of Weld Metals

**Figure 10:**
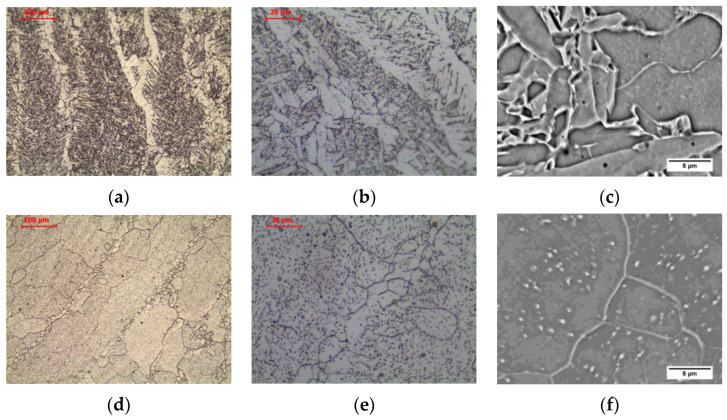
Microstructure of weld metal: (**a**–**c**) as-welded state; (**d**–**f**) after long-term exposure at 600 °C, LM-BF, and SEM-BSE.

The microstructure in weld area heated above the liquidus temperature is characterized by a typical casting structure (Figure 10a–c). The lack of a clear dendritic structure is due to the fact that the partition coefficient k is close to 1, which prevents micro-segregation of alloying elements into interdendritic spaces. The second reason is the phase transformations that occur during the cooling of the weld. The material that has been melted and recrystallized undergoes a complete transformation into austenite and then into ferrite with pearlite by eutectoid transformation. In the microstructure of the weld, primary ferrite (allotriomorphic ferrite, polygonal ferrite, grain boundary ferrite), side-plate ferrite in the Widmanstatten pattern, and fine-plate ferrite (acicular ferrite) were observed. A ferrite that nucleates at the boundary of a columnar austenite grain and grows into the grain is called a grain boundary ferrite due to the fact that it does not have a regular faceted shape reflecting its internal crystalline structure. Because of its presence in the structure, it is possible to approximate the size of former columnar austenite grains. At lower temperatures, the mobility of the flat ferrite growth front at the grain boundary decreases, and Widmanstatten ferrite (side-plate ferrite) is formed instead. At even lower temperatures, new acicular ferrite nucleates on the inclusion particles and has randomly oriented short ferrite needles with the basket weave feature. These observations are confirmed in the references [6,31,36,37].

Long-term exposure of weld area at 600 °C, as in the previous zones, led to a change in the form of cementite (Figure 10d–f). Globular precipitates of iron carbide in the ferritic matrix are observed. The structure of acicular ferrite and Widmanstatten ferrite completely disappears. The region of the polygonal ferrite is a zone completely or partially free of carbide precipitates, while in the region of the former acicular and Widmanstatten ferrites, a large amount of spheroidite is visible. Non-metallic inclusions present in the weld are revealed, and subboundaries of columnar grains on which polygonal ferrite nucleation has not occurred are also visible.

### 3.4. Microstructure of Heat-Affected Zones in Weld Metals

**Figure 11:**
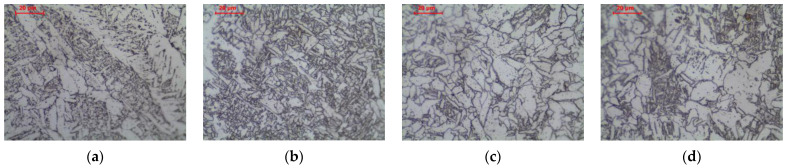
Microstructure of welded joint (as-welded): (**a**) weld metal; (**b**) intercritical HAZ; (**c**) fine grain HAZ; (**d**) coarse grain HAZ, LM-BF.

**Figure 12:**
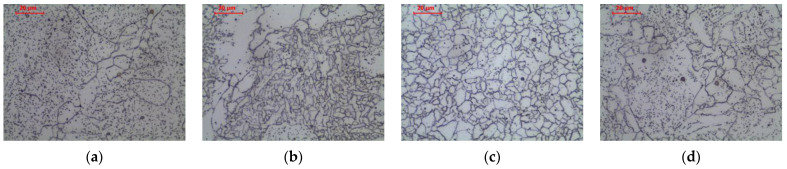
Microstructure of welded joint (as-welded and long-term exposure at 600 °C): (**a**) weld metal; (**b**) intercritical HAZ; (**c**) fine grain HAZ; (**d**) coarse grain HAZ, LM-BF.

The welding thermal cycle of the weld also significantly affects the microstructure of the previously made weld, which, similarly to the basic material, is heated to different maximum temperatures depending on the distance from the fusion line of the next bead. Figure 11 and Figure 12 show the microstructure of the weld metal and three basic zones in its HAZ in an as-welded state and after long-term exposure at 600 °C, which are described in detail below.

Multi-pass welding is characterized by the influence on the welded material and previously made welds by a multiple thermal welding cycle. Each subsequent pass heats the previous stitches to different temperatures [30,38,39,40]. This results in the formation of HAZ areas in the previous welds similar to those observed in the base material (Section 3.2). Due to the fact that the phase transition α → γ during heating causes fragmentation of the grains of the previous cast weld structure, their size will be finer than that of the original weld structure. This has a positive effect on the plastic properties of the weld, especially the impact strength.

**Figure 13:**
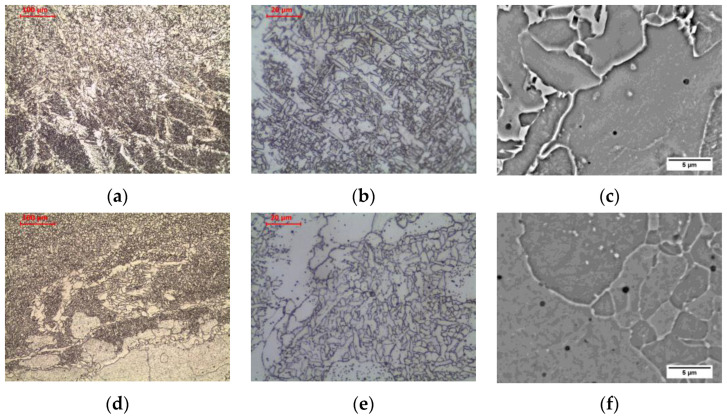
Microstructure of intercritical heat-affected zone in weld: (**a**–**c**) as-welded state; (**d**–**f**) after long-term exposure at 600 °C, LM-BF and SEM-BSE.

In the weld heated in ICHAZ area, the structure is partially recrystallized. The presence of polygonal, side-plate, and acicular ferrites not transformed as a result of the thermal cycle, as well as allotriomorphic ferrite after transformation, was observed (Figure 11b and Figure 13a–c). Long-term exposure of ICHAZ area of the weld at 600 °C led to cementite spheroidization, as before (Figure 12b and Figure 13d–f).

**Figure 14:**
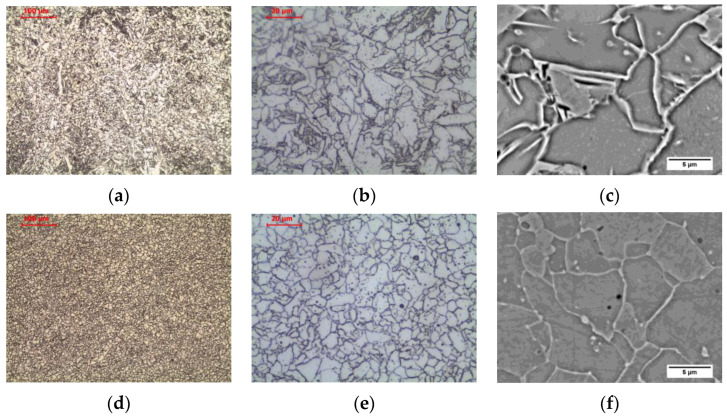
Microstructure of fine-grain heat-affected zone in weld: (**a**–**c**) as-welded state; (**d**–**f**) after long-term exposure at 600 °C, LM-BF, and SEM-BSE.

In the weld heated to FGHAZ area, the structure is completely recrystallized. The columnar crystal structure of the weld basically disappears, and the grain structure is observed in the normalized state. Allotriomorphic ferrite was observed after transformation (Figure 11c and Figure 14a–c). Long-term exposure of FGHAZ area of the weld at 600 °C led to the obtaining of a microstructure (Figure 12c and Figure 14d–f) that corresponds to the structure observed in the HAZ of the base material (Figure 5c).

**Figure 15:**
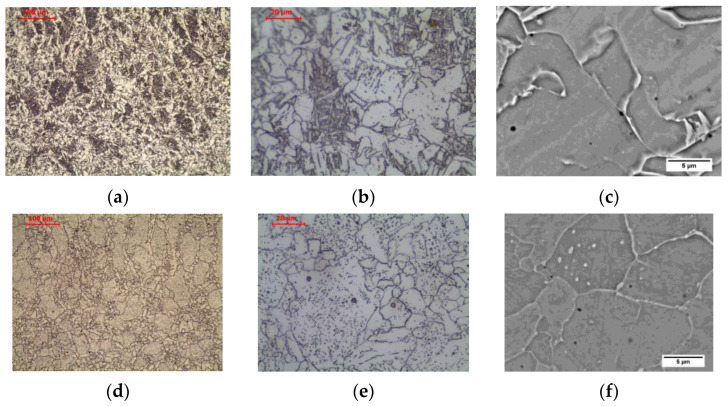
Microstructure of coarse-grain heat-affected zone in weld: (**a**–**c**) as-welded state; (**d**–**f**) after long-term exposure at 600 °C, LM-BF, and SEM-BSE.

In the weld heated in CGHAZ area, the structure is completely recrystallized, and due to the high temperature of the heat cycle of welding the next pass, the growth of the austenite grain was impacted. In the structure, the occurrence of allotriomorphic ferrite, nucleating on the grain boundaries of former austenite, as well as side-plate ferrite and acicular ferrite, can be observed (Figure 11d and Figure 15a–c). Long-term exposure of CGHAZ area of the weld at 600 °C led to a ferritic microstructure with globular carbides (Figure 12d and Figure 15d–f), with allotriomorphic ferrite distinguished in the structure nucleating on the boundaries of former austenite grains, as well as ferrite in the area of former side-plate and acicular ferrite.

## 4. Conclusions

Microscopic analysis of the welded joint of unalloyed steel in the as-welded condition and in the condition after long-term exposure to the temperature of 600 °C showed a significant impact of both factors on the microstructure.

- The thermal cycle of welding leads to a heat-affected zone of coarse grain (CGHAZ), fine grain (FGHAZ), and uneven grain (ICHAZ), with the band structure inherited from the base material being retained in each case. Prolonged exposure at 600 °C leads to a change in the form of cementite in pearlite from lamellar to spheroidal in each of the zones, constantly maintaining the band structure.
- In the case of the weld in the as-welded condition, polygonal, side-plate, and acicular ferrites are observed. As a result of long-term exposure to a temperature of 600 °C, cementite assumes a spherical shape, while side-plate and acicular ferrite disappear.
- Microscopic examination of the weld in the area of influence of the next pass on the previous one reveals the heat-affected zone in which partial (ICHAZ) and complete (FGHAZ) recrystallization of the structure and the formation of new allomorphic ferrite grains occur. The CGHAZ area, which also undergoes complete recrystallization, has a structure composed of the three types of ferrite (polygonal, side-plate, acicular), of which the side-plate and acicular ferrite disappear after prolonged exposure at 600 °C.

## Figures and Tables

**Table 1:** Chemical composition of base materials and weld, % mass.

Data Source	C	Si	Mn	P	S	N	Cu
OES (steel)	0.123	0.183	1.14	0.0135	0.0046	-	0.027
EN 10025-2	≤0.170	-	≤1.40	≤0.035	≤0.035	≤0.012	≤0.55
OES (weld metal)	0.0743	0.524	1.24	0.0161	0.0057	-	0.050
EN ISO 14341-A	0.06–0.14	0.5–0.8	0.9–1.3	≤0.025	≤0.035	-	≤0.35

## Data Availability

Not applicable.
